# Recent advances in percutaneous coronary intervention for modernizing coronary physiology-guided and device-based strategies for coronary revascularization

**DOI:** 10.3389/fmed.2026.1790673

**Published:** 2026-04-30

**Authors:** Daowen Tang, Wenji Mu

**Affiliations:** Department of Cardiology, Gucheng District People’s Hospital, Lijiang, Yunnan, China

**Keywords:** complex PCI, coronary artery disease, coronary physiology, drug-eluting stents, intravascular imaging, percutaneous coronary intervention, revascularization strategies

## Abstract

Coronary artery disease (CAD) remains a leading cause of morbidity and mortality worldwide. Over the past three decades, percutaneous coronary intervention (PCI) has evolved from a anatomical revascularization technique into a precision-based, physiology- and imaging-guided therapeutic strategy capable of addressing increasingly complex coronary disease. This review aims to critically examine contemporary advances in PCI, with particular emphasis on the integration of coronary physiology, intravascular imaging, and next-generation device technologies. We synthesize evidence from landmark randomized trials, meta-analyses, and recent high-impact studies to evaluate how these advances have reshaped clinical decision-making, procedural optimization, and long-term outcomes in complex coronary artery disease. A comprehensive narrative review of the literature was conducted using PubMed, Google Scholar, and the National Library of Medicine databases. The review focuses on physiology-guided PCI, drug-eluting stent evolution, intravascular imaging, PCI in complex clinical settings (including left main disease and post-coronary artery bypass grafting anatomy), and emerging adjunctive technologies. Structural interventions are discussed selectively to contextualize the broader evolution of catheter-based cardiovascular therapies. Contemporary PCI has been transformed by physiology-guided lesion assessment, advanced intravascular imaging, and improved stent platforms, enabling safer and more effective treatment of complex coronary anatomy. Evidence from recent trials highlights both the strengths and limitations of physiology-guided PCI, particularly in multivessel and post-CABG disease, underscoring the need for integrated anatomy-physiology approaches. Advances in device engineering, robotic assistance, and adjunctive pharmacotherapy have further refined procedural precision and outcomes. Emerging technologies, including drug-coated balloons, resorbable scaffolds, and innovative biologic and cell-free therapeutic strategies, illustrate the expanding translational landscape of coronary intervention. The modernization of PCI reflects a paradigm shift toward precision-guided, evidence-based coronary revascularization. While technological advances have broadened the scope of PCI and improved procedural outcomes, optimal revascularization strategies must remain individualized and grounded in robust clinical evidence.

## Highlights

- PCI has evolved into a precision-based strategy integrating coronary physiology, imaging, and advanced device technologies.
- Physiology-guided PCI improves lesion selection but has limitations in complex and post-CABG disease.
- Advances in drug-eluting stents, intravascular imaging, and adjunctive technologies have expanded PCI indications.
- Evidence-based, anatomy- and physiology-driven decision-making remains essential for optimal revascularization.
- Emerging device and biologic innovations highlight future directions in coronary intervention.

## 1 Introduction

Coronary artery disease (CAD) is one of the significant causes of morbidity and mortality globally, imposing significant health and economic burdens on developed countries. In the United States alone, CAD is responsible for approximately 790,000 heart attacks each year (1), contributing to an economic impact estimated at $89 billion in 2016, projected to soar to $215 billion by 2035 (2). The pursuit of biodegradable scaffolds began with Keiji Igaki and Hideo Tamai’s polylactide stent in 2000. Despite technical challenges, long-term follow-up showed minimal complications. The development of drug-eluting bioresorbable scaffolds, such as the everolimus-eluting ABSORB A device, demonstrated favorable outcomes with low adverse cardiovascular events like CAD and reduced luminal compromise compared to metallic stents (3). These advancements indicate a promising future for bioresorbable vascular scaffolds in interventional cardiology (4). In summary, the history of coronary interventions is marked by significant advancements and continuous innovation. From the pioneering work of Andreas Grüentzig to the development of drug-eluting as well as bioresorbable stents, each milestone has contributed to improved patient outcomes and the evolution of interventional cardiology (4–6).

For the past a few years, advancements in therapeutic approaches over the past decade, which have reduced CAD-related mortality and improved survival rates post-myocardial infarction, the prevalence of CAD is expected to rise due to an aging population (7). This context explores importance of continuing to refine and develop new strategies for CAD management (8–10). The advent of PCI has revolutionized the treatment landscape for CAD. PCI has been a focal point of intensive research and development, leading to significant improvements in procedural outcomes and patient survival. For the past a few years, research has emerged supporting the integration of coronary physiology into angiography to guide PCI decisions. For instance, fractional flow reserve (FFR) has been pivotal in providing physiological guidance, with extensive clinical trials validating its utility. Despite clinical guidelines recommending FFR for most patients with coronary stenosis, its adoption remains suboptimal in routine practice (11).

To enhance the application of physiology-guided interventions, non-hyperemic coronary pressure measurements have been introduced. These non-hyperemic indices offer a practical alternative to FFR, enabling more widespread use in clinical settings. A few more reports have focused on developing and validating various non-hyperemic pressure ratios. Furthermore, previous reports also explored the fundamental principles of coronary physiology under non-hyperemic conditions, the rationale for using these measurements to assess stenosis, and the current evidence supporting their clinical application. Additionally, it addresses the discordance between non-hyperemic ratios and FFR and highlights the significance of these clinical parameters for developing suitable treatment regimen for CAD and other cardiovascular ailments (11). In the recent years, research on interventional cardiology is advancing, including new guidelines on revascularization, innovative treatments for cardiogenic shock, and novel stent technologies. The new European Society of Cardiology (ESC) guidelines pertinent to myocardial revascularization provide updated evidence-based recommendations, emphasizing the crucial implications non-invasive and invasive tools for ischemia assessment. The guidelines also highlight the strategy of culprit-vessel-only revascularization in cardiogenic shock, which has demonstrated favorable outcomes at 12 months, challenging the necessity of bystander-vessel revascularization (12–18).

Furthermore, innovations in stent technologies and clinical trials in stent technologies, particularly from Asia, have shown non-inferior results compared to established stents. However, randomized trials for absorbable scaffolds revealed satisfactory clinical outcomes but an enhanced incidence of stent thrombosis compared to conventional stents, without the anticipated reduction in angina. Furthermore, data indicating no benefit from discontinuing aspirin post-stenting have prompted a re-evaluation of long-standing practices. The importance of the heart team in revascularization decisions is crucial for immediate diagnostic angiography in clinically appropriate cases of acute ST-segment elevation myocardial infarction (STEMI). Provisional stenting for bifurcation lesions has been upgraded to a Class I recommendation, while absorbable scaffolds are not recommended outside clinical trials. The findings from the CULPRIT SHOCK trial have influenced guidelines, emphasizing the benefit of treating only the culprit vessel in patients with myocardial infarction, multivessel disease, and cardiogenic shock (19). The future of coronary interventions integrating advanced diagnostic and therapeutic modalities, including virtual AI-based technologies, to enhance treatment precision and outcomes. Personalized medicine approaches, leveraging pharmacogenomics and patient-specific factors, are essential for optimizing therapy. Combining cell-based therapies with traditional treatments may offer synergistic benefits, while long-term monitoring and ethical considerations will ensure the sustainability and acceptance of new therapies. International collaboration and technological advancements in bioengineering will drive the next generation of coronary interventions, aiming to improve patient care globally (20).

The continuous evolution of coronary interventions, guided by technological and clinical evidence, holds promise for significantly improving the management of CAD. By embracing innovative strategies and collaborative efforts, the field of interventional cardiology is poised to achieve remarkable strides in enhancing patient outcomes and addressing the global burden of heart disease. In this review, we discussed coronary physiology, intravascular imaging, and next-generation device-based PCI technologies and various other dual therapies along with virtual AI based technologies in diagnosis, various clinical trials and existing therapeutic modalities ameliorating the heart diseases by coronary interventions.

### 1.1 Literature search

A structured narrative review of the literature was performed using PubMed, Google scholar, and the National Library of Medicine databases. Peer-reviewed articles, consensus documents, and landmark clinical trials published in English were comprehensively screened. The review prioritizes advances in physiology-guided PCI, the evolution of drug-eluting stent platforms, intravascular imaging modalities, and PCI strategies in complex clinical scenarios, including left main coronary artery disease and post–coronary artery bypass graft anatomy. Emerging adjunctive technologies such as robotic assistance, intracoronary diagnostics, and digital integration tools were also evaluated for their clinical relevance. Structural and transcatheter interventions were included selectively to provide contextual insight into the broader progression of catheter-based cardiovascular therapies, without deviating from the primary focus on coronary revascularization.

## 2 Evolution and periodic trendline of PCI

**FIGURE 1:**
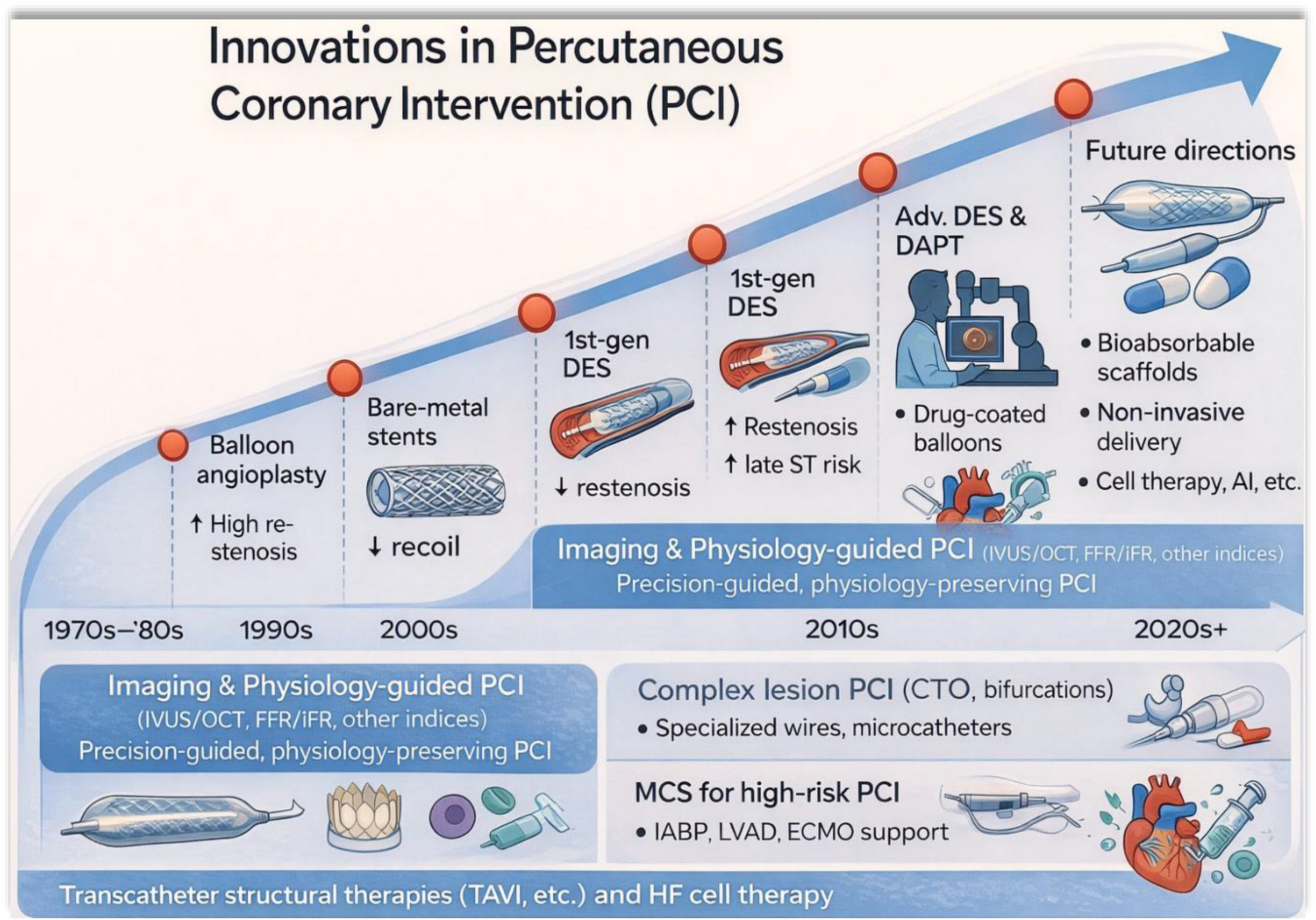
The figure illustrates the chronological development of PCI from the 1970s to the present. Early revascularization strategies began with balloon angioplasty, which was limited by high restenosis rates. The introduction of bare-metal stents (BMS) in the 1990s reduced vessel recoil, followed by first-generation drug-eluting stents (DES) in the 2000s that significantly lowered restenosis but raised concerns regarding late stent thrombosis. Subsequent advancements include improved DES platforms, optimized dual antiplatelet therapy (DAPT), and drug-coated balloons for selected lesions. Contemporary PCI integrates intravascular imaging and physiology-guided approaches such as intravascular ultrasound (IVUS), optical coherence tomography (OCT), and fractional flow reserve/instantaneous wave-free ratio (FFR/iFR) to enable precision-guided revascularization. The figure also highlights complex lesion interventions, mechanical circulatory support for high-risk PCI, and emerging future directions including bioabsorbable scaffolds, non-invasive therapeutic delivery systems, artificial intelligence-assisted strategies, and regenerative cell-based therapies.

PCI has evolved substantially over the past five decades through progressive technological and physiological innovations. Early revascularization strategies in the 1970s–1980s were dominated by balloon angioplasty, which was limited by high restenosis rates. The introduction of bare-metal stents (BMS) in the 1990s reduced vessel recoil and improved procedural outcomes. In the early 2000s, first-generation drug-eluting stents (DES) significantly lowered restenosis but were associated with concerns regarding late stent thrombosis. Subsequent advancements in the 2010s led to improved DES platforms, optimized dual antiplatelet therapy (DAPT), and the development of drug-coated balloons for selected lesions. Concurrently, the integration of intravascular imaging and physiology-guided strategies, including intravascular ultrasound (IVUS), optical coherence tomography (OCT), and fractional flow reserve (FFR/iFR), has enabled precision-guided PCI (Figure 1). Modern practice also incorporates complex lesion interventions such as chronic total occlusions and bifurcation lesions using specialized guidewires and microcatheters, often supported by mechanical circulatory support devices in high-risk PCI. Looking forward, emerging technologies such as bioabsorbable scaffolds, non-invasive therapeutic delivery, artificial intelligence-assisted interventions, and regenerative cell-based therapies are expected to further transform coronary revascularization strategies.

## 3 Current multiple implications of coronary interventions in heart diseases

**FIGURE 2:**
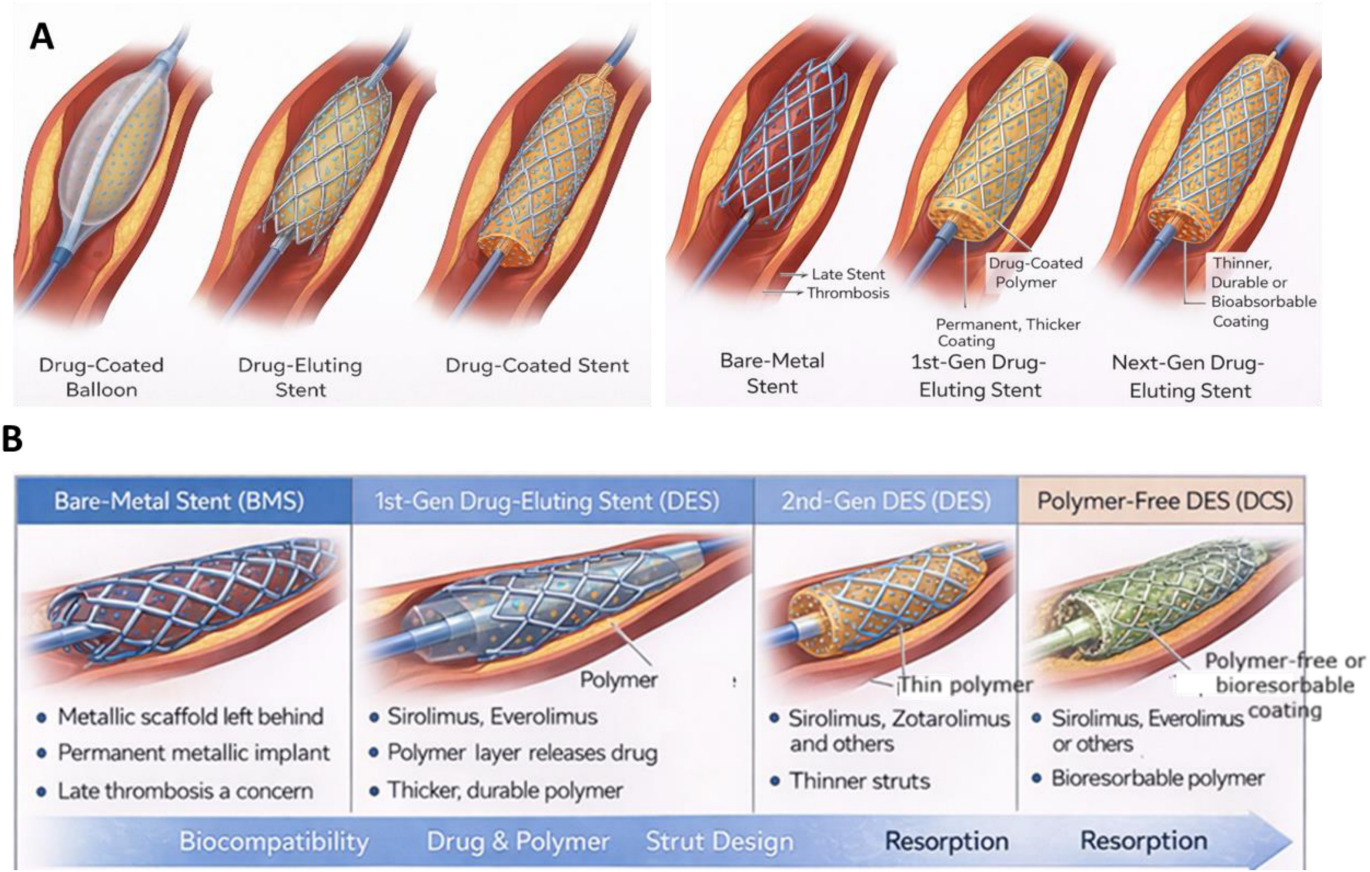
Mechanisms of drug delivery and evolution of stent design in percutaneous coronary intervention (PCI). **(A)** Upper panel illustrates the mechanisms of drug delivery in contemporary PCI devices, including drug-coated balloons (DCB), drug-eluting stents (DES), and drug-coated stents (DCS). Drug-coated balloons deliver antiproliferative agents directly to the vessel wall during balloon inflation without leaving a permanent scaffold. Drug-eluting stents provide mechanical support while releasing antiproliferative drugs from a polymer-coated metallic platform over time. Drug-coated stents represent a modified stent platform in which the drug is applied directly to the stent surface, often using polymer-free or thin coating technologies. **(B)** The lower panel depicts the evolution of coronary stent design, beginning with bare-metal stents (BMS), followed by first-generation drug-eluting stents, second-generation DES with thinner struts and improved biocompatible polymers, and polymer-free or bioresorbable coating technologies. These advancements aim to improve vascular healing, reduce restenosis and thrombosis risk, and enhance long-term clinical outcomes in contemporary PCI practice.

PCI with drug-eluting stent (DES) implantation has emerged as the preferred revascularization strategy for the majority of patients experiencing acute coronary syndromes (ACS). Thus, advancements in both devices and techniques, stent thrombosis remains the most significant short-term complication following PCI, particularly in the context of ACS (21–24) (Figures 1, 2). For the patients with the progression of CAD, traditionally, the saphenous vein graft (SVG) was the considered as conduit of choice; however, arterial grafts such as left as well as right internal thoracic arteries and radial artery were reported with a greater patency rates (21–25). Despite notable advancements, secondary revascularization frequently becomes necessary, with PCI being the preferred approach following coronary artery bypass graft (CABG) surgery (21–25). The procedural nuances and clinical outcomes for PCI in this patient subset markedly differ from those without prior CABG due to altered coronary anatomy and conduit pathophysiology. Notably, PCI in saphenous vein grafts carries a higher risk of complications, prompting operators to emphasize embolic protection strategies and favor complex interventions on native vessels. Often, SVGs are utilized as conduits to facilitate native-vessel PCI rather than being targeted directly (21–25). Previous reports also described the distinct pathophysiological changes in conduits, advancements in CABG surgical techniques, and contemporary evidence surrounding PCI in patients with prior CABG. However, several future reports are required to explore how these factors impact procedural safety and long-term efficacy, with a particular emphasis on strategies to mitigate complications and optimize clinical outcomes (21–25).

### 3.1 Conduit pathophysiology and advancements in CABG surgery

The pathophysiology of graft conduits, particularly SVGs, evolves significantly over time post-CABG, affecting their susceptibility to atherosclerosis and thrombosis. SVGs tend to develop diffuse intimal hyperplasia and atheroma, which can lead to occlusion and necessitate revascularization (25). This increased propensity for adverse events underscores the importance of embolic protection devices during PCI in SVGs to reduce the risk of distal embolization and no-reflow phenomenon. Recent advancements in CABG techniques, such as the use of arterial grafts (e.g., internal mammary artery) instead of SVGs, have demonstrated improved patency rates and reduced long-term complications (25). Enhanced surgical methods, including off-pump CABG and minimally invasive approaches, have also contributed to better clinical outcomes and reduced perioperative morbidity (25). PCI Strategies Post-CABG are beneficial. In patients with previous CABG, PCI strategies must be tailored to address the unique challenges presented by graft and native vessel pathophysiology. For native vessel PCI, operators often encounter more complex lesions due to diffuse disease progression and prior interventions (25). Techniques such as advanced imaging modalities (e.g., intravascular ultrasound and optical coherence tomography) and novel stent technologies (e.g., drug-eluting stents with bioresorbable polymers) have improved procedural success and long-term outcomes (25).

When addressing SVG lesions, the use of embolic protection devices is crucial to minimize the risk of embolization. Additionally, drug-coated balloons and newer generation DES have shown promise in reducing restenosis rates and improving graft patency. The decision to perform PCI on native vessels versus SVGs should be guided by factors such as graft viability, lesion complexity, and overall patient health (25). Clinical outcomes for PCI in patients with prior CABG are influenced by a multitude of factors, including the type of graft used, the time elapsed since CABG, and the presence of comorbid conditions. Studies have shown that PCI in this population is associated with higher rates of restenosis and adverse cardiac events compared to those without prior CABG. However, with meticulous patient selection and the use of advanced techniques and devices, the long-term efficacy of PCI can be significantly improved (25). Emerging evidence supports the use of hybrid revascularization strategies, combining CABG and PCI to optimize outcomes. This approach leverages the durability of arterial grafts for critical vessels while utilizing PCI for more accessible or less critical vessels, offering a tailored intervention to complex coronary artery disease. In conclusion, secondary revascularization with PCI in patients with previous CABG presents unique challenges and opportunities. Understanding the pathophysiological changes in graft conduits, incorporating advanced surgical and interventional techniques, and adopting a patient-centered approach are pivotal for improving safety and long-term efficacy. Future research should continue to explore innovative strategies and technologies to further enhance the care and outcomes of this complex patient population (25).

Furthermore, the field of cardiac cell therapy has faced significant challenges due to variable cardiac pathophysiology. By understanding the mechanisms more clearly and obtaining promising clinical results, the potential for cardiac cell therapy is gradually being revitalized (26). Carotid artery stenting (CAS) and carotid endarterectomy (CEA) have demonstrated similar outcomes for asymptomatic patients with significant carotid artery stenosis, as highlighted by the ACST-2 trial. This study involved patients with severe unilateral or bilateral carotid artery stenosis who required intervention and were randomly assigned to undergo either CAS or CEA, with a follow-up period extending over 5 years. The trial findings indicated that the risk of disabling stroke or death during the periprocedural period was approximately 1%, with an annual incidence rate of 0.5% in the subsequent 5 years. There were no significant differences in the risk of stroke or death between the two treatment groups (27). These results align with data from other comparative trials of CAS and CEA, providing robust evidence that both procedures offer similar risks and benefits for both asymptomatic and symptomatic patients with carotid artery stenosis (27).

Comparative Efficacy of CAS and CEA: The ACST-2 trial’s findings, along with results from other studies, elucidate that both CAS and CEA are effective in managing carotid artery stenosis with comparable safety profiles. This is particularly relevant for asymptomatic patients where the decision to intervene is often guided by the potential risks of stroke. The similarity in outcomes between CAS and CEA allows for personalized treatment choices based on patient-specific factors such as anatomical considerations, comorbid conditions, and patient preference (27). Advancements in both CAS and CEA techniques have contributed to improved safety and efficacy. In CAS, the development of better stent designs and embolic protection devices has reduced the risk of perioperative complications such as stroke. Similarly, refinements in CEA, including the use of minimally invasive approaches and enhanced perioperative care, have also improved patient outcomes (27). The long-term follow-up in the ACST-2 trial and other studies indicates that both CAS and CEA provide durable protection against stroke. The consistent annual rate of 0.5% for disabling stroke or death beyond the periprocedural period highlights the effectiveness of these interventions in preventing recurrent cerebrovascular events. This is particularly significant for asymptomatic patients, where the primary goal of intervention is to prevent future strokes rather than address immediate symptoms (27).

For clinicians, the decision to choose CAS or CEA should be individualized, taking into account the patient’s overall health status, vascular anatomy, and potential procedural risks. For instance, CAS might be preferred in patients with higher surgical risks or those with contraindications to general anesthesia. Conversely, CEA might be more suitable for patients with complex aortic arch anatomy or heavily calcified lesions that could complicate stent placement (27). Ongoing research and clinical trials continue to refine the indications and techniques for CAS and CEA. Emerging technologies such as drug-eluting stents, bioresorbable scaffolds, and improved imaging modalities for procedural planning and follow-up are poised to enhance the outcomes of carotid revascularization further. Additionally, studies focusing on the role of pharmacotherapy in conjunction with revascularization could provide insights into optimizing long-term management strategies for patients with carotid artery disease (27). In conclusion, both CAS a viable option for the management of severe carotid artery stenosis, offering similar safety and efficacy profiles. The choice between these procedures should be tailored to the individual patient’s clinical scenario and preferences. As advancements in technology and procedural techniques continue to evolve, the outcomes of carotid revascularization are expected to improve further, providing better protection against stroke and enhancing the quality of life for patients with carotid artery disease (27).

### 3.2 Deferred angiography in non-ST-Elevation OHCA

The TOMAHAWK study, a multicenter trial, has demonstrated that immediate coronary angiography does not lower the 30-day mortality rate in patients experiencing out-of-hospital cardiac arrest (OHCA) without ST-segment elevation when compared to a delayed angiography strategy (28). This finding is consistent with results from the COACT trial, which similarly found no significant difference in clinical outcomes between immediate and delayed angiography at 90 days and 1 year. It is crucial to note that while the COACT trial only included patients with shockable arrest rhythms, the TOMAHAWK trial expanded its cohort to include patients with both shockable and non-shockable rhythms. Notably, the TOMAHAWK trial revealed that the combined secondary endpoint of death or severe neurological deficit was more frequent in patients receiving immediate angiography. However, the researchers advise interpreting this outcome with caution due to potential statistical concerns related to multiple testing applications for Clinical Practice (28). These findings have significant implications for clinical practice. Immediate angiography has traditionally been favored under the assumption that it would promptly identify and treat coronary occlusions, thereby improving survival and neurological outcomes. However, the evidence from TOMAHAWK and COACT suggests that this approach does not confer a mortality benefit and may, in fact, be associated with worse neurological outcomes in some cases. This challenges the conventional paradigm and suggests that a more selective approach to angiography timing based on individual patient risk factors and arrest characteristics may be warranted (28).

For many years, bleeding was considered an unavoidable trade-off for strategies aimed at minimizing thrombotic complications in patients undergoing PCI. However, recent shifts in clinical paradigms recognize bleeding as a serious prognostic factor, comparable to new or recurrent ischemic or thrombotic events. This has led to increased focus on developing strategies that enhance both the efficacy and safety of antithrombotic treatments and procedural techniques before, during, and after PCI. Innovations in antithrombotic therapy, such as the development of novel anticoagulants and antiplatelet agents with more favorable bleeding profiles, are being actively pursued (28). Research should aim to refine patient selection criteria for immediate versus delayed angiography in OHCA, taking into account the type of arrest rhythm and other clinical factors that may influence outcomes. Additionally, further studies are needed to explore the optimal balance between preventing thrombotic events and minimizing bleeding risk in PCI. This includes investigating new antithrombotic agents and procedural innovations that could offer better safety profiles without compromising efficacy (28). The TOMAHAWK trial has significantly influenced the understanding of the timing of angiography in OHCA patients, challenging previously held beliefs about the benefits of immediate intervention. Concurrently, the evolving perspective on bleeding in PCI highlights the importance of safety alongside efficacy in antithrombotic strategies. As research continues, these insights will guide more nuanced and effective clinical practices, ultimately improving patient outcomes in both emergency and elective cardiac care settings (29).

## 4 Clinical perspectives of other coronary interventions: future aspects

The increasing frequency of PCI in patients with prior CABG surgery is notable in contemporary catheterization laboratories (30). PCI is often preferred over redo surgery for bypass grafts despite higher acute and long-term event rates than PCI pertinent to the native vessels (31). Bypass graft interventions are particularly challenging due to the advanced age and multiple comorbidities of these patients, as well as the complexity and thrombotic nature of the stenoses. Angiographic assessment of stenosis severity in bypass conduits is more difficult than in the native arteries, making the suitability of PCI crucial (32), especially for intermediate, equivocal stenoses (32, 33). This is essential to avoid exposing patients to higher procedural risks particularly in multivessel disease (34). FFR-guided PCI related to native coronary stenoses is resulted in the enhanced long-term health outcomes in clinical settings (20, 35–37). A recent retrospective registry study has examined the long-term clinical outcomes of patients undergoing FFR-guided PCI compared to angiography-guided PCI for intermediate stenoses in bypass grafts. The study’s results demonstrate that FFR-guided PCI is both safe and more effective than angio-guided PCI, particularly for intermediate stenoses in bypass grafts. Advantages of FFR-guided PCI were especially notable in arterial grafts, where it significantly reduced procedural costs and lowered the incidence of periprocedural myocardial infarction (PMI) in saphenous vein grafts. These findings suggest that using FFR to guide PCI in intermediate stenoses can lead to improved patient outcomes and more efficient resource utilization (20). However, PCI of bypass grafts is associated with adverse effects than PCI pertinent to native vessels (32), which primarily due to greater rates of PMI as well as the repeat revascularizations. Stenoses related to the bypass grafts are typically due to degenerated or thrombotic plaques, which pose a greater risk of acquiring atherothrombotic embolization. To mitigate distal embolization, strategies such as proximal as well as the distal embolic protection devices have been employed, typically mitigating PMI rates (38). However, these devices were infrequently used in patients treated with angio-guided revascularization in the registry, possibly contributing to greater rates of PMI among the individuals in angio-guided group (39). This elucidates the limitations of the treatment strategy, highlighting the necessity of FFR guidance in mitigating PCIs executed in the venous grafts, thereby preventing PMI (35–37, 40–42).

FFR-guided PCI has shown favorable outcomes in various clinical and, but its use in intermediate stenoses of bypass grafts had not been well-documented until this study. The findings extend previous evidence and have significant clinical implications, considering the difficulty in assessing residual myocardial ischemia in stable patients who has prior CABG. FFR, with its superior spatial resolution, overcomes the limitations pertinent to the non-invasive functional tests during the multivessel disease (34, 41, 43–48). Despite technical challenges in performing FFR assessments in bypass grafts, the study demonstrated that deferring PCI of intermediate stenoses based on negative FFR values is safe; the clinical outcomes out of these procedures are comparatively better than the angio-guided strategy.

Future research should focus on refining FFR techniques and developing more advanced embolic protection devices to further reduce PMI rates. Additionally, long-term studies comparing FFR-guided and angio-guided strategies in diverse patient populations could provide more comprehensive insights into the best practices for managing complex coronary interventions. In the context of transcatheter aortic valve implantation (TAVI) related to severe aortic stenosis, recent findings from NOTION-3 trial described at ESC Congress 2024 indicate that PCI reduces the incidence of significant adverse effects among the patients with stable CAD compared to conservative treatment. Aortic stenosis and CAD share risk factors, etiology, and clinical presentation, with approximately 50% of TAVI patients also having CAD. However, current European and US guidelines do not provide clear recommendations related to the execution of PCI in addition to TAVI, suggesting an area for future guideline development and clinical investigation (20). The integration of FFR guidance in PCI for bypass grafts and the exploration of PCI in TAVI patients represent significant innovation are essential to refine these techniques and improve patient outcomes, ultimately leading to more effective and safer cardiovascular care (49).

## 5 Dual therapies along with coronary interventions

Platelets could modulate inflammatory processes that lead to vascular atherosclerosis (50, 51). There is a direct correlation between platelet aggregability and systemic atherosclerotic disease (52). Myocardial infarction (MI), a severe manifestation of coronary atherosclerosis, is primarily driven by the activation of platelets (53). Platelet activity plays a crucial role in the development and progression of vascular atherosclerosis, as highlighted by several studies (50, 51, 54). In patients with stable CAD, there is a notable increase in platelet reactivity and higher levels of circulating monocyte-platelet aggregates, which serve as early indicators of acute MI (55, 56). Additionally, platelet reactivity tends to enhance with the involvement of multiple vascular territories affected by atherosclerosis, including cerebral, cardiac, and peripheral regions (52, 57). The relationship between the extent of coronary atherosclerosis and platelet reactivity post-clopidogrel administration was significant, as evidenced by the number of diseased vessels and the total stent length (TSL). This suggests that more extensive coronary artery involvement is associated with increased platelet activity, even after antiplatelet therapy, highlighting the need for tailored therapeutic strategies in patients with diffuse coronary atherosclerosis (57). Consequently, several therapeutic strategies to modulate aggregability of platelets are developed.

For example, combinatorial regimen of aspirin and clopidogrel is typically used antiplatelet regimen in patients who are subjected to PCI (58). However, residual high platelet reactivity post-administration of clopidogrel is related to the higher acquisition of cardiovascular events (59–61). Despite these insights, the relationship between residual platelet reactivity post-clopidogrel and their effects on coronary atherosclerosis severity yet require more studies. Furthermore, a previous report has examined whether higher residual platelet reactivity could partly explain adverse periprocedural outcomes in patients reported with coronary atherosclerosis (57).

Coronary manipulation and side branch occlusion could be typical risk factors for periprocedural MI during PCI (62). Multiple reports established a direct link between the number and total length of stents implanted and adverse periprocedural outcomes (63–65). Patients associated with MVD are at greater risk of acquiring periprocedural myonecrosis due to more extensive coronary manipulation. Suboptimal platelet inhibition, indicated by the higher rate of HPR in MVD patients, likely contributes to this increased risk. HPR was identified as the significant predictor pertinent to periprocedural MI, consistent with previous research (59, 66). Aggressive platelet inhibition, such as with eptifibatide, has been shown to mitigate periprocedural MI risk in patients with high TSL to levels seen in patients with lower TSL (57, 65).

Future studies should focus on refining antiplatelet therapies and personalizing treatment strategies based on genetic, cellular, and clinical factors influencing platelet reactivity. Exploring novel agents or combination therapies that can more effectively inhibit platelet activity without increasing bleeding risk is crucial. Emerging technologies like advanced imaging and genetic testing could help better identify patients at risk of suboptimal platelet response to clopidogrel, allowing for more tailored interventions. Additionally, machine learning algorithms could predict platelet reactivity patterns, improving patient outcomes. Conducting large-scale, multi-center clinical trials to validate these findings and develop comprehensive guidelines for managing patients with high platelet reactivity undergoing PCI is essential. These guidelines should incorporate individualized treatment plans and advanced diagnostic tools to optimize patient care. By integrating these approaches, the management of coronary interventions can be significantly enhanced, leading to improved clinical outcomes and reduced cardiovascular event rates.

## 6 Device-based innovations in contemporary PCI

The accompanying figure summarizes the mechanisms of drug delivery and the technological evolution of coronary stent platforms in contemporary PCI (67–71). Modern PCI strategies employ several device-based approaches to inhibit neointimal proliferation and maintain vessel patency. Drug-coated balloons (DCB) enable rapid transfer of antiproliferative agents to the arterial wall during balloon inflation without leaving a permanent scaffold, thereby preserving vascular physiology and facilitating future interventions. In contrast, drug-eluting stents (DES) combine mechanical vessel scaffolding with controlled release of antiproliferative drugs from polymer-based coatings, which has significantly reduced restenosis rates compared with earlier devices (67–71). Drug-coated stents (DCS) represent a further refinement in which therapeutic agents are applied directly to the stent surface using polymer-free or ultrathin coating technologies designed to improve vascular compatibility. Parallel to these advances in drug delivery, coronary stent design has evolved from bare-metal stents to first-generation DES, followed by second-generation platforms characterized by thinner struts, improved alloys, and more biocompatible or bioresorbable polymers (67–71). Collectively, these technological innovations aim to enhance endothelial healing, minimize restenosis and late stent thrombosis, and improve long-term clinical outcomes in contemporary PCI practice (67–71) (Figure 2 and Table 1).

**TABLE 1:** Comparative Characteristics of drug-coated Balloons, drug-eluting stents, and drug-coated stents in contemporary PCI.

Parameter	Drug-coated balloon (DCB)	Drug-eluting stent (DES)	Drug-coated stent (DCS)	References
Implanted scaffold	No permanent implant	Permanent metallic scaffold	Metallic scaffold with surface drug coating	(67–71)
Drug delivery mechanism	Antiproliferative drug delivered during balloon inflation	Sustained drug release from polymer-coated stent	Drug applied directly on stent surface, often polymer-free	(67–71)
Drug release duration	Rapid transfer during balloon contact	Controlled release over weeks to months	Intermediate drug release depending on coating	(67–71)
Common drugs used	Paclitaxel (most common)	Sirolimus, everolimus, zotarolimus, biolimus	Sirolimus- or paclitaxel-based coatings	(67–71)
Primary mechanism of restenosis prevention	Inhibition of smooth muscle proliferation without scaffold	Mechanical scaffolding plus sustained antiproliferative drug release	Scaffolding combined with surface drug delivery	(67–71)
Typical clinical indications	In-stent restenosis, small vessel disease, selected *de novo* lesions	Majority of *de novo* coronary artery lesions; complex PCI	Selected lesions where reduced polymer exposure is desirable	(67–71)
Vessel scaffolding	None	Strong scaffolding preventing recoil	Moderate scaffolding with drug coating	(67–71)
Vessel healing profile	Preserves natural vessel physiology	Possible delayed endothelialization	Potentially improved endothelial healing	(67–71)
Risk of restenosis	Low in selected lesions with adequate lesion preparation	Very low with contemporary DES	Low but device-dependent	(67–71)
Risk of thrombosis	Very low (no permanent implant)	Low with new-generation DES but still present	Potentially reduced polymer-related thrombosis	(67–71)
Dual antiplatelet therapy (DAPT)	Often shorter duration	Standard or longer duration	Potentially shorter depending on device	(67–71)
Advantages	No permanent implant, preserves vessel anatomy, future treatment options remain open	Strong evidence base, excellent acute procedural success	Reduced polymer exposure, potential improvement in vascular healing	(67–71)
Limitations	Requires optimal lesion preparation; risk of recoil or dissection	Permanent metallic implant; risk of late stent failure or neoatherosclerosis	Less clinical evidence compared with DES	(67–71)
Typical clinical role	Niche but expanding indications	Current gold standard for most PCI procedures	Emerging alternative strategy	(67–71)

### 6.1 Strategies for dual antiplatelet therapy (DAPT) in acute coronary syndromes conventional DAPT and associated risks

Standard DAPT for patients who are diagnosed with acute coronary syndromes receiving PCI typically includes aspirin and a potent P2Y12 inhibitor, such as prasugrel or ticagrelor, for a duration of 12 months. While this regimen effectively reduces ischemic risks, it also significantly increases the risk of bleeding (72). To mitigate bleeding risks, strategies such as de-escalation of DAPT intensity or abbreviation of DAPT duration are employed. This approach is particularly beneficial for patients with high bleeding risk and without high ischemic risk, as it reduces bleeding without increasing ischemic events (72).

Despite the absence of direct head-to-head clinical trials comparing these strategies, an international expert panel has summarized the evidence and provided guidance on assessing ischemic and bleeding risks. The Consensus Statement aims to help clinicians optimize DAPT approaches, improving patient outcomes by balancing efficacy and safety. Current European and North American guidelines on managing ST-segment elevation myocardial infarction (STEMI) (22, 73, 74), non-ST-segment elevation ACS (21, 75), and PCI (73, 76) offer limited guidance on antithrombotic therapy options (77). Innovations in cardiovascular interventions, such as drug-eluting resorbable scaffolds and tailored antiplatelet therapy strategies, offer significant advancements in patient care. By integrating these novel approaches, healthcare providers can improve clinical outcomes for patients with complex coronary and peripheral artery diseases, ensuring a balance between efficacy and safety.

### 6.2 Clinical implications of drug-eluting resorbable scaffolds and angioplasty

For patients suffering from chronic limb-threatening ischemia (CLTI) due to infrapopliteal artery disease, treatment with everolimus-eluting resorbable scaffolds has been shown to provide superior clinical outcomes compared to angioplasty. This significant finding was highlighted by the LIFE-BTK trial, presented at the TCT 2023 conference.

### 6.3 LIFE-BTK trial design and findings

LIFE-BTK trial involved CLTI patients comorbid with the infrapopliteal artery disease. Participants were assigned to receive either an everolimus-coated resorbable scaffold or undergo angioplasty. and the results related to this study demonstrated a clear advantage of the resorbable scaffold over angioplasty to foster enhanced clinical outcomes (72).

### 6.4 Optical coherence tomography (OCT) in PCI: divergent outcomes in clinical trials

Usage of OCT to guide PCI has yielded conflicting evidence from two clinical trials. For instance, the OCTOBER trial reported improved clinical outcomes in patients with complex coronary artery bifurcation lesions when OCT guidance was used. Conversely, the ILUMIEN IV trial, which involved patients who are at greater risk of acquiring coronary artery lesions, did not find a similar benefit (78).

## 7 Evolution of Robotic -PCI

While advancements in percutaneous coronary intervention have significantly improved patient outcomes (79–86), the working conditions for interventional cardiologists in the cardiac catheterization laboratory have remained largely unchanged since the introduction of coronary angioplasty. Efforts to enhance these working conditions have led to the development of robotic-assisted PCI (R-PCI) systems.

Mechanism and efficacy of R-PCI: CorPath GRX system, a pioneering technology developed by Corindus Vascular Robotics and currently utilized in the USA, features an innovative design comprising a bedside unit and an interventional cockpit. The bedside unit, which is strategically placed on the procedure table, includes a single-use sterile cassette, an articulating arm, and a sophisticated robotic drive. This cassette is manually loaded with 0.014-inch guidewires, as well as rapid exchange angioplasty as well as stent delivery systems, which are subsequently mounted on the robotic drive. The system’s control is executed remotely from the interventional cockpit, enabling precise manipulation of the devices during coronary procedures. Percutaneous Robotically-Enhanced Coronary Intervention (PRECISE) study marks the first large-scale, multicenter trial to assess the safety and efficacy of a novel robotic system for PCI (87). This significant study enrolled 164 patients diagnosed with angiographically confirmed obstructive CAD and documented myocardial ischemia. R-PCI procedures were conducted using the CorPath 200 system. According to previous studies, R-PCI is a feasible and safe alternative to traditional manual PCI (M-PCI), showcasing its potential in enhancing the precision and outcomes of coronary interventions. The study’s robust design and extensive patient cohort provided substantial evidence supporting the clinical application of robotic assistance in PCI. The results elucidate the technological advancements in interventional cardiology, highlighting the potential of robotic systems to improve procedural accuracy, reduce operator fatigue, and potentially enhance patient outcomes. This innovative approach to coronary intervention represents a significant step forward in the integration of robotics into clinical practice, paving the way for future developments in the field.

**FIGURE 3:**
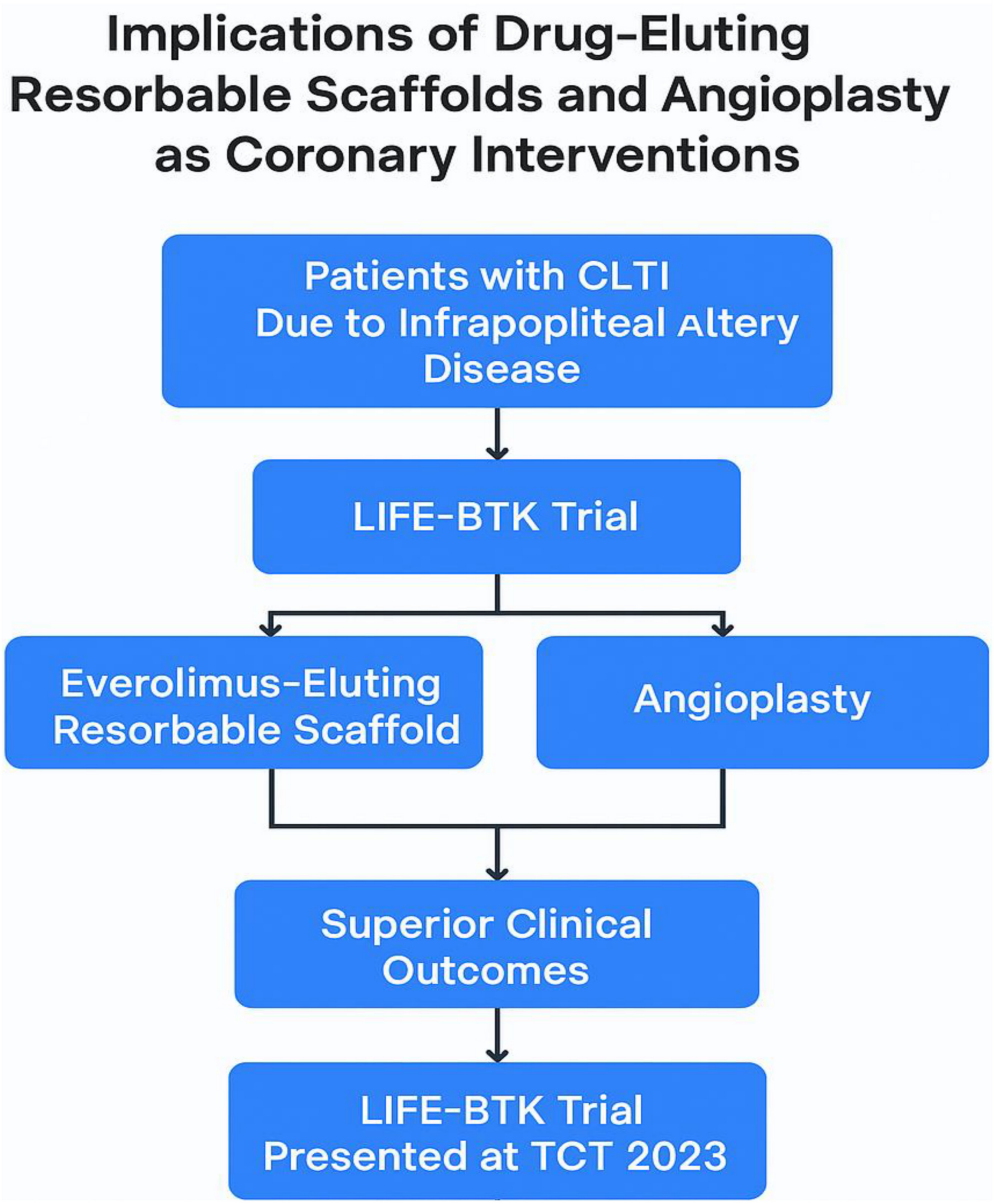
Flowchart illustrating the implications of drug-eluting resorbable scaffolds versus angioplasty in the management of chronic limb-threatening ischemia (CLTI) due to infrapopliteal artery disease. This diagram summarizes the LIFE-BTK trial design and its clinical relevance. Patients with CLTI were randomized to receive either an everolimus-eluting resorbable scaffold or standard angioplasty. The flowchart highlights the superior clinical outcomes associated with the scaffold intervention, as demonstrated by the findings presented at the TCT 2023 conference. This visual emphasizes the therapeutic advantage and evolving interventional strategies in peripheral arterial disease.

Additionally, studies have shown that R-PCI is as safe and effective as M-PCI in treating coronary disease. However, for R-PCI to gain widespread acceptance, it must offer tangible benefits over M-PCI for both patients and operators. One significant advantage of robotic PCI is the substantial reduction in procedural radiation exposure for the operator. Interventional cardiologists are exposed to the highest levels of radiation among healthcare professionals, and reducing this exposure can mitigate long-term health risks, including radiation-induced cancers and cataracts (Figure 3).

Thus, R-PCI could be implicated to use against complex coronary lesions. Despite this, patients with ST-elevation myocardial infarction (STEMI) have been excluded from most R-PCI trials, representing a significant gap in the evidence. Addressing this gap is crucial for the broader adoption of R-PCI in cardiac catheterization laboratories. The ongoing TREAT GRX study is a prospective, single-arm, multicenter observational study designed to ascertain CorPath GRX system efficiency in a real-world setting, including patients with complex CAD. R-PCI represents a promising advancement in interventional cardiology, offering clear benefits in reducing radiation exposure. Additionally, the concept of tele-stenting performing PCI remotely holds exciting potential but will require overcoming significant technical and logistical challenges. Upto 10% of R-PCI cases may need partial manual intervention, the overall technical success rates are reasonable, even for more complex CAD cases (88).

Furthermore, applications of virtual reality (VR) technology for cardiac interventions have been gaining medical significance. Virtual reality is a rapidly advancing technology that holds significant promise for medical applications. The integration of VR into daily clinical settings may enhance the robustness, patient-centeredness, and safety of clinical care as part of coronary interventions (89). Future research should focus on expanding the capabilities of robotic systems to handle a broader range of interventional devices and techniques, including those needed for highly calcified lesions and bifurcation procedures. Additionally, further studies are needed to evaluate the efficacy and safety of R-PCI in acute settings, such as STEMI, to ensure that these systems can be safely and effectively used in all patient populations. The integration of advanced imaging techniques, like OCT, with robotic systems may also enhance the precision and outcomes of PCI procedures (88).

## 8 PCI implications in left main CAD

In 1976, the VA Cooperative Trial demonstrated a survival benefit of bypass surgery over optimal medical therapy for unprotected left main coronary artery disease (ULMT) (90). This, combined with the initial poor and unpredictable results of early balloon angioplasty (91, 92), established bypass surgery as standard treatment for ULMT, leading to a strong discouragement of PCI in this context (93, 94). Several significant advancements have enhanced PCI efficacy for unprotected left main trunk (ULMT) since 1990s. These include enhanced antiplatelet therapies, the implications of intravascular ultrasound, and the development of drug-eluting stents. Additionally, optimal medical therapy, particularly statin use, has become a critical component of treatment. Encouraging results from recent registries provided the impetus for randomized trials to determine the optimal treatment for ULMT. The SYNTAX (Synergy Between Percutaneous Coronary Intervention With Taxus and Cardiac Surgery) trial, with its 2-year follow-up results, offers valuable insights into the current state of knowledge (95).

Registries and randomized clinical trials (RCTs) both play essential roles in evaluating treatment outcomes, but each has limitations. Outcomes pertinent to this registry describe real-world outcomes after physicians choose the suitable therapeutic regimen for the individual patient, leading to inherent differences between patients who received PCI or bypass surgery (96).

RCTs, on the other hand, often have limited external validity because the recruited patients may not represent the typical clinical population, often being younger and with fewer comorbidities. Despite this, RCTs are crucial for establishing treatment benefits, which can then be assessed for broader applicability through subsequent registry analyses (88). Future research should aim to bridge the gap between RCTs and real-world practice by including more diverse patient populations in trials and ensuring that sample sizes are adequate to provide conclusive results. Additionally, the development of personalized treatment approaches recognizing that “one size does not fit all” is paramount. New technologies such as advanced imaging techniques and bioresorbable scaffolds may further enhance the efficacy and safety of PCI for unprotected left main trunk. Continuous advancements in antiplatelet therapy and the integration of precision medicine approaches will likely play significant roles in optimizing patient outcomes. In summary, while bypass surgery has historically been the standard treatment for ULMT, significant advancements in PCI techniques and technologies have made it a viable alternative for many patients. Ongoing research and clinical trials are essential to refine these approaches, ensure their safety and efficacy, and expand their applicability to a broader range of patients (88).

## 9 Novel perspectives on cardiology interventions: insights from DES and BMS meta-analysis

A previous report (81) conducted a comprehensive meta-analysis that compared drug-eluting stents (DES) and bare-metal stents (BMS) among the patients who were undergoing PCI, with follow-up extending up to 3 years. The study included both crude and adjusted data, highlighting the potential for confounding factors and selection bias. The investigators cautioned against direct comparison of overall rates between BMS and DES, yet reported similar 6- to 12-month mortality rates between the two. Notably, DES significantly reduced the need for target vessel or lesion revascularization. By the 3-year mark, DES demonstrated superior outcomes in terms of mortality, myocardial infarction, and target vessel or lesion revascularization. Another study (82) provided additional insights with a 5-year follow-up study from an Italian center, in which the clinical outcomes were compared after PCI and bypass surgery. In this report, PCI was linked to a mitigated incidence of combined adverse outcomes such as death, MI, or stroke, whereas bypass surgery demonstrated a lower rate of target vessel revascularization. There was no significant difference in cardiac death rates between the two treatments, but a nearly 6% incidence of stent thrombosis was observed. Patients who received therapy for complex CAD, particularly those who represent the “last bastion” need open heart surgery, remains a contentious issue among surgeons and interventional cardiologists. This group is characterized by unique anatomical challenges, and their treatment has significant implications for the future of cardiac revascularization strategies.

To improve treatment strategies for this complex patient group, a robust knowledge base is essential. More research is needed to determine which patients will benefit most from each revascularization strategy. Data from Kim et al. (97) and other studies must be synthesized to develop clearer guidelines. Long-term outcomes and safety: While current data suggest that DES have better long-term outcomes compared to BMS, ongoing research should continue to ascertain safety as well as the efficacy pertinent to DES over extended follow-up periods. Innovation in stent technology: The development of new stent technologies, such as bioresorbable scaffolds and next-generation drug-eluting stents, could further improve outcomes and reduce complications like stent thrombosis. Personalized medicine approaches: Personalized treatment regimen depending on the individual patient characteristics, such as genetic profiling and risk factor analysis, could optimize the choice between PCI and bypass surgery. Collaborative research efforts: Multicenter, international collaborations are crucial for collecting comprehensive data and developing consensus guidelines. These efforts will help ensure that treatment strategies are based on the best available evidence (88).

## 10 Other advancements in PCI technology

PCI has seen remarkable advancements since the initial use of balloon angioplasty to dilate coronary stenosis. The introduction of bare-metal stents marked a significant improvement, providing predictable luminal gain and mitigating vessel dissection and recoil. However, bare-metal stents were associated with complications like stent thrombosis and in-stent restenosis, which were addressed through the development of DAPT to reduce thrombosis rates to below 1%, and the advent of drug-eluting stents that lowered restenosis rates to single digits (20). The first-generation drug-eluting stents, while effective, showed a late thrombosis signal, necessitating extended DAPT durations. Subsequent generations of drug-eluting stents with thinner struts and improved pharmacological agents were introduced, yielding favorable outcomes. Unlike earlier trials that often involved selected, low-risk patients, recent studies have included more diverse and complex patient populations to better reflect real-world scenarios.

### 10.1 Innovations and clinical trials

TARGET All Comers trial (16) is particularly notable as it rigorously tested the Firehawk stent when compared to the established Xience stent from Abbott Vascular in a European setting. Despite the high incidence of complex lesions and acute coronary syndromes (even in the STEMI) in trial cohort, both stents demonstrated low 12-month target-lesion failure rates (6.1% for Firehawk and 5.9% for Xience). The trial met its non-inferiority endpoint, suggesting that the new technology, featuring thin struts and an absorbable abluminal polymer, performed on par with current DES. The long-term benefits of these innovations, particularly the absence of a polymer, are still under investigation. Further technical enhancements, such as even thinner stent struts, are anticipated to incrementally improve long-term outcomes post-PCI.

## 11 Emerging and Under-represented domains in contemporary PCI

### 11.1 Drug-coated balloons (DCBs): expanding the paradigm beyond permanent implants

Drug-coated balloons (DCBs) have emerged as a transformative PCI technology, particularly in clinical scenarios where permanent metallic implantation is undesirable. Contemporary evidence highlights their utility in in-stent restenosis (ISR), small-vessel disease, bifurcation lesions, and increasingly in *de novo* coronary lesions. Recent analyses demonstrate that DCB-based strategies achieve comparable target lesion revascularization (TLR) rates to second-generation drug-eluting stents (DES), while significantly reducing the risk of late stent thrombosis and preserving native vascular physiology (98). Long-term follow-up data further suggest that DCB-only approaches may limit chronic inflammation and neoatherosclerosis associated with permanent implants (99). Recent consensus described that optimized lesion preparation, intravascular imaging guidance, and appropriate drug transfer kinetics are critical determinants of procedural success (100). As such, DCBs are increasingly regarded not merely as niche devices, but as integral components of a “leave-nothing-behind” PCI philosophy.

### 11.2 Chronic total occlusion (CTO) PCI: from technical feasibility to prognostic benefit

CTO PCI represents one of the most technically demanding yet rapidly advancing areas in coronary intervention. While earlier studies focused primarily on procedural success, recent investigations emphasize patient-centered outcomes, including symptom relief, quality of life, arrhythmia reduction, and long-term survival. Large observational cohorts and meta-analyses demonstrate that successful CTO recanalization is associated with improved angina status, enhanced left ventricular function, and superior quality-of-life metrics compared with optimal medical therapy alone (101). Importantly, emerging data suggest that CTO PCI may also reduce arrhythmic burden and long-term mortality in selected patient populations (102). Technological advancements such as hybrid algorithms, dedicated CTO guidewires, microcatheters, and dual-injection imaging have markedly improved procedural success rates while maintaining acceptable safety profiles, even in elderly or high-risk cohorts (103). These findings position CTO PCI as a key component of modern revascularization strategies rather than an optional or purely symptomatic intervention.

### 11.3 Mechanical circulatory support (MCS) devices in high-risk PCI and cardiogenic shock

**FIGURE 4:**
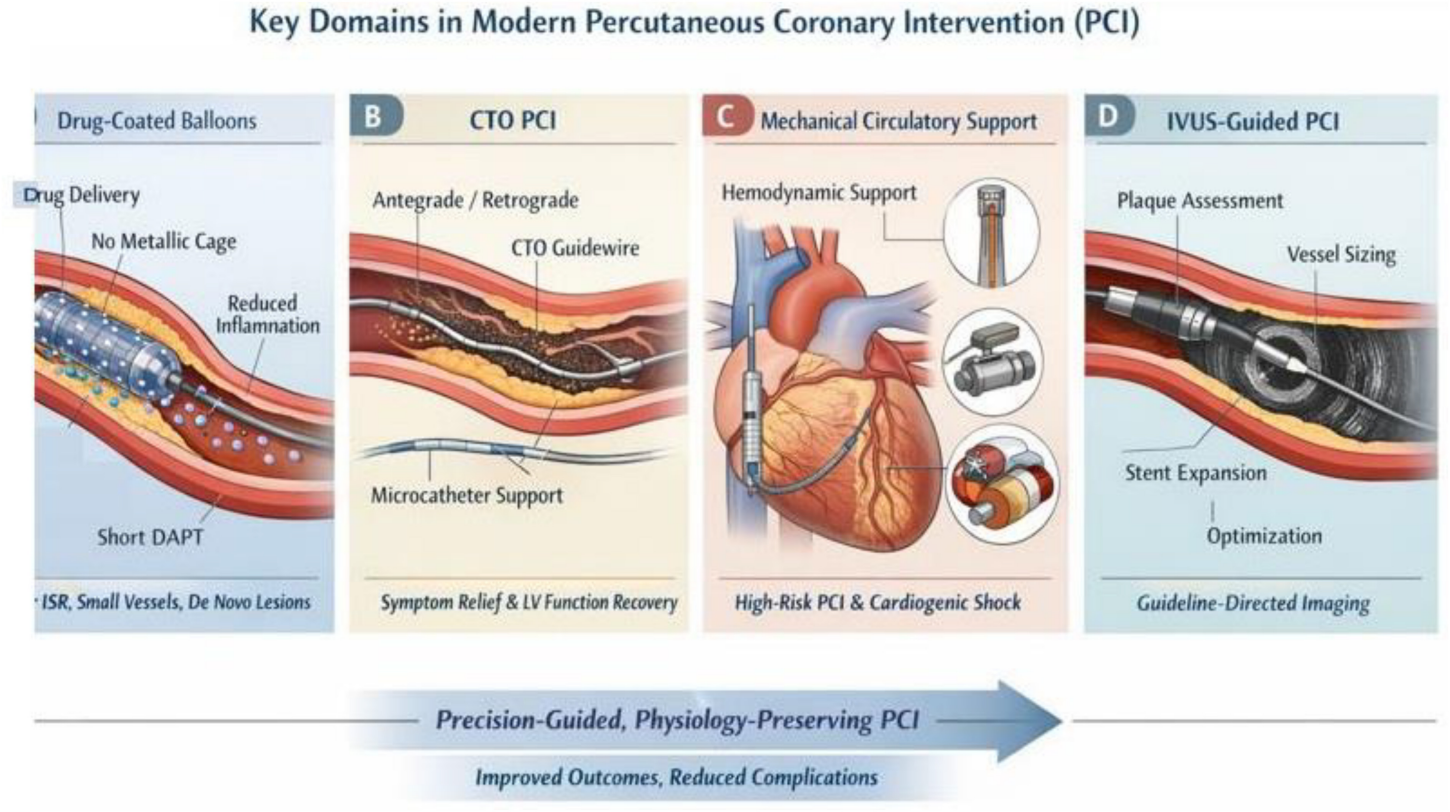
Key domains in modern percutaneous coronary intervention (PCI). Schematic overview of four under-represented yet critical domains shaping contemporary PCI practice. **(A)** Drug-coated balloons (DCBs) enable effective coronary revascularization without permanent metallic implantation, facilitating homogeneous antiproliferative drug delivery, reduced chronic inflammation, and shortened dual antiplatelet therapy, particularly in in-stent restenosis, small-vessel disease, and selected *de novo* lesions. **(B)** Chronic total occlusion (CTO) PCI illustrates advanced antegrade and retrograde strategies using dedicated guidewires and microcatheters to restore distal coronary flow, resulting in symptomatic relief and improvement in left ventricular function. **(C)** Mechanical circulatory support (MCS) devices, including percutaneous ventricular assist systems, provide hemodynamic stabilization during high-risk PCI and cardiogenic shock, enabling safer and more complete revascularization in complex coronary anatomy. **(D)** Intravascular ultrasound (IVUS)-guided PCI highlights the role of intravascular imaging in plaque characterization, accurate vessel sizing, and optimization of stent expansion and apposition, thereby improving procedural precision and long-term clinical outcomes. Collectively, these advances support a transition toward precision-guided, physiology-preserving PCI with improved patient outcomes and reduced procedural complications.

Temporary mechanical circulatory support (tMCS) devices including intra-aortic balloon pump (IABP), Impella, TandemHeart, and veno-arterial extracorporeal membrane oxygenation (VA-ECMO) have become increasingly integrated into contemporary PCI practice, particularly for high-risk PCI and acute myocardial infarction-related cardiogenic shock (Figure 4).

Recent randomized trials and individual-patient meta-analyses reveal that while routine use of active MCS has not consistently demonstrated a mortality benefit, early, selective deployment in hemodynamically unstable patients may improve procedural safety and end-organ perfusion (104). However, these benefits must be weighed against device-related complications, including bleeding, vascular injury, and stroke (105). Current consensus statements emphasize the importance of patient selection, timing of support initiation, and multidisciplinary shock team models to optimize outcomes and minimize harm (106). As device technology and evidence mature, MCS is increasingly viewed as an adjunct to enable complex PCI rather than a universal therapeutic solution.

### 11.4 Intravascular ultrasound (IVUS): from adjunctive tool to guideline-mandated standard

The IVUS warrants dedicated emphasis given its central role in contemporary PCI. IVUS provides high-resolution, cross-sectional imaging that enables precise assessment of lesion morphology, vessel size, plaque burden, and stent expansion. Recent expert consensus demonstrate that IVUS-guided PCI significantly reduces target vessel failure, stent thrombosis, and cardiac mortality, particularly in complex lesions such as left main disease, long lesions, and true bifurcations (107). Consequently, the 2024 ESC guidelines upgraded IVUS- or OCT-guided PCI to a Class I, Level A recommendation for anatomically complex coronary disease (108, 109). Beyond procedural optimization, IVUS has prognostic implications, with operator experience and imaging guidance independently associated with improved long-term survival outcomes (110). These findings firmly establish intravascular imaging as a cornerstone rather than an adjunct of precision-based coronary intervention. Collectively, recent high-impact evidence elucidates that drug-coated balloons, CTO PCI, mechanical circulatory support devices, and intravascular imaging are integral to the modernization of PCI. Their thoughtful integration into clinical practice enhances procedural safety, expands revascularization options, and aligns coronary intervention with precision-medicine principles. Incorporating these domains into the review would strengthen its completeness, align it with current guideline-directed practice, and better reflect the rapidly evolving landscape of contemporary coronary revascularization.

### 11.5 Bioresorbable vascular scaffolds implications in coronary interventions

The development of fully bioresorbable vascular scaffold (BVS) Absorb is considered as a major advancement in coronary revascularization. Designed to provide temporary scaffolding that would dissolve over time, leaving a restored vessel, early clinical evaluations were promising. However, the hypothesis that BVS would reduce angina more effectively than stents was constrained by safety concerns, particularly an increased rate of scaffold thrombosis.

The ABSORB IV trial (17), which included 2,604 patients randomly assigned to receive either the Absorb BVS or a standard everolimus-eluting stent, aimed to demonstrate non-inferiority in efficacy at 30 days as well as at one year, along with potential angina relief. The primary endpoint of target-lesion failure at 30 days was observed in 5.0% pertinent to Absorb BVS group versus 3.7% in everolimus-eluting stent group. At 1 year, target-lesion failure rates were 7.8 and 6.4%, respectively, while angina incidence at 1 year was similar between the groups (20.3% vs. 20.5%).

Although these results met non-inferiority criteria, and the minimal clinical outcomes over existing DES and the higher thrombosis rate led to the discontinuation of the Absorb BVS (20) (Table 2).

**TABLE 2:** Recent clinical trials in coronary interventions.

Trial name	Patients inclusions	Trial results	Implication of trial in PCI	References
CULPRIT SHOCK	Patients with myocardial infarction, multivessel disease, and cardiogenic shock	Treating only the culprit vessel in myocardial infarction patients resulted in better outcomes.	Influenced guidelines to recommend focusing on the culprit vessel only in cardiogenic shock patients.	(19)
ACST-2	Patients with severe unilateral or bilateral carotid artery stenosis	Similar outcomes for CAS and CEA in terms of disabling stroke or death during the periprocedural period. Annual incidence of 0.5% disabling stroke or death in the following five years.	Demonstrated that both CAS and CEA are effective for managing carotid artery stenosis, allowing personalized treatment choices based on patient-specific factors.	(27)
TOMAHAWK	Patients with out-of-hospital cardiac arrest (OHCA) without ST-segment elevation	No significant difference in 30-day mortality between immediate and delayed angiography. Higher incidence of death or severe neurological deficit in immediate angiography group.	Challenges the conventional practice of immediate angiography for OHCA patients, suggesting a more selective approach based on individual patient risk factors.	(28)
NOTION-3	Patients undergoing TAVI with stable CAD	PCI reduces incidence of significant adverse effects compared to conservative treatment in TAVI patients.	Highlights the potential benefits of combining PCI with TAVI in patients with severe aortic stenosis and CAD, suggesting future guideline updates are needed.	(20)
LIFE-BTK	Patients with chronic limb-threatening ischemia (CLTI) and infrapopliteal artery disease	Everolimus-eluting resorbable scaffolds provide superior clinical outcomes compared to angioplasty.	Supports the use of resorbable scaffolds over angioplasty in patients with CLTI and infrapopliteal artery disease.	(72)
OCTOBER	Patients with complex coronary artery bifurcation lesions	Improved clinical outcomes with OCT-guided PCI.	Suggests the benefit of OCT guidance in PCI for complex bifurcation lesions, potentially improving procedural precision and patient outcomes.	(78)
ILUMIEN IV	Patients at high risk of coronary artery lesions	No significant benefit of OCT guidance in high-risk patients.	Indicates that OCT guidance may not offer additional benefits in all patient groups, emphasizing the need for selective use based on lesion complexity and patient risk.	(78)
TARGET All Comers	Patients with complex lesions and acute coronary syndromes	Both Firehawk and Xience stents showed low 12-month target-lesion failure rates, meeting non-inferiority endpoint.	Demonstrates that new stent technologies with absorbable polymers perform comparably to established DES, paving the way for further innovations in stent design.	(16)
ABSORB IV	Patients with coronary artery disease undergoing PCI	Non-inferiority in efficacy at 30 days and one year for Absorb BVS compared to standard everolimus-eluting stents, but higher thrombosis rate with BVS.	Highlights the need for cautious use of BVS in clinical practice due to safety concerns, guiding future improvements in bioresorbable scaffold technology.	(17)

This table summarizes the latest clinical trials in coronary interventions, providing insights into the evolving strategies and technologies aimed at optimizing patient outcomes in various cardiovascular conditions. It highlights the implications of these trials on clinical practice and future guideline development. CAD, Coronary artery disease; PCI, Percutaneous coronary intervention; CAS, Carotid artery stenosis; DES, Drug-eluting stents; OCT, Optical coherence tomography; TAVI, Transcatheter aortic valve implantation.

### 11.6 Significant highlights of the above trials

- *CULPRIT SHOCK trial*: Emphasizes the benefit of treating only the culprit vessel in myocardial infarction patients with cardiogenic shock.
- *ACST-2 trial*: Demonstrates that both CAS and CEA are effective for managing carotid artery stenosis, allowing personalized treatment choices.
- *TOMAHAWK trial:* Challenges the conventional practice of immediate angiography for OHCA patients, suggesting a more selective approach.
- *LIFE-BTK trial*: Supports the use of everolimus-eluting resorbable scaffolds over angioplasty in patients with chronic limb-threatening ischemia.
- *ABSORB IV trial:* Highlights the need for cautious use of bioresorbable vascular scaffolds in clinical practice due to safety concerns.

The evolution of PCI technologies continues to hold promise for further reducing complications and improving patient outcomes. The integration of coronary physiology into routine practice, particularly through non-hyperemic pressure ratios, offers potential for more accurate and widespread use of physiological guidance in PCI. Additionally, ongoing research into new stent materials, designs, and bioresorbable technologies aims to enhance the long-term success of coronary interventions. As the field progresses, it will be essential to continue rigorous clinical evaluations and adopt a personalized approach to treatment, leveraging advancements in pharmacogenomics and patient-specific factors. The collaboration between international researchers and clinicians is crucial in driving these innovations forward, ultimately improving the care and prognosis for patients with CAD.

Hence, comparative analysis of DES and BMS, along with insights from long-term follow-up studies, describes the evolving landscape of cardiology interventions. As the field progresses, it is vital to continue refining revascularization strategies, incorporating new technologies, and personalizing treatment approaches to improve patient outcomes. Collaborative research and a robust knowledge base will be essential in addressing the challenges and advancing the practice of interventional cardiology (88).

### 11.7 Transcatheter treatment of pure aortic regurgitation: clinical implications

The early application of transcatheter aortic valve implantation (TAVI) for treating non-calcific aortic regurgitation revealed significant concerns regarding its short-term efficacy. Unlike calcific aortic stenosis, where the calcified leaflets provide anchorage for the valve, non-calcific aortic regurgitation lacks these stabilizing structures, leading to potential complications such as valve displacement or paravalvular leakage. Understanding valve-anatomy interactions: A critical factor in improving TAVI outcomes for aortic regurgitation is a comprehensive understanding of the interactions between the transcatheter heart valves and the unique anatomical features of patients with non-calcific aortic valve disease. These patients present distinct challenges due to the absence of calcification, which typically aids in the secure placement of the valve.

Advances with dedicated TAVI devices: Recent advancements have introduced TAVI devices manufactured in order to foster non-calcific aortic regurgitation. These dedicated devices feature enhanced anchoring mechanisms and sealing properties, tailored to address the unique challenges posed by this condition. This innovation marks a significant step forward, offering new opportunities for effective management of aortic regurgitation (111).

As TAVI technology evolves, it is crucial to refine patient selection criteria to identify those who will benefit most from these specialized devices. Comprehensive risk stratification can help predict procedural success and long-term outcomes. Further research and development in valve design should focus on customization based on individual anatomical variations. This approach could enhance the fit and function of transcatheter valves in diverse patient populations. While short-term outcomes have been the focus, long-term data is essential to evaluate the durability and effectiveness of dedicated TAVI devices for non-calcific aortic regurgitation. Ongoing studies and registries should aim to provide robust long-term evidence. Utilizing advanced imaging modalities include 3D echocardiography and computed tomography can improve pre-procedural planning and intra-procedural guidance, leading to better alignment and positioning of the transcatheter valves. Effective management of aortic regurgitation using TAVI requires a multidisciplinary approach, involving cardiologists, cardiac surgeons, imaging specialists, and interventionalists. Collaborative decision-making can optimize patient outcomes. Incorporating patient preferences and quality of life considerations into treatment decisions is vital. Shared decision-making models that involve patients in the planning process can lead to better satisfaction and adherence to treatment plans. As new devices and techniques are introduced, ongoing training and skill development for healthcare providers are essential. Specialized training programs can ensure that interventionalists are proficient in the latest advancements.

Evolution of TAVI for non-calcific aortic regurgitation represents a significant advancement in cardiology interventions. By addressing the unique challenges of this condition with dedicated devices and comprehensive patient management strategies, the field can achieve better procedural success and improved long-term outcomes. Continued innovation, research, and multidisciplinary collaboration will be key to unlocking the full potential of TAVI in treating aortic regurgitation (111).

### 11.8 Advancements and challenges in cell therapy as coronary interventions for heart failure

Heart failure (HF) remains a major global health challenge, responsible for 13% of deaths globally. Prognosis pertinent to HF patients is grim, with a 50% overall survival within five years of diagnosis. A critical issue in ischemic HF is the irreversible loss of cardiomyocytes, coupled with the heart’s limited regenerative capacity. Currently, heart transplantation is the only definitive treatment for replacing damaged heart muscle. Emerging cell therapy could be a hopeful strategy to foster cardiac repair through tissue regeneration, despite significant obstacles in both preclinical and clinical stages. Ongoing clinical trials that investigate cell-based as well as cell product-based therapies for treating HF. These trials are essential for translating regenerative cardiac therapies into clinical practice. They evaluate the safety and efficacy of various cell-based treatments and explore innovative treatment protocols, including recurrent intravenous dosing, personalized patient selection using pharmacogenomics, different methods of cell delivery (112).

For instance, clinical trials currently are examining the efficacy related to various novel cell types, including cardiomyocytes derived from pluripotent stem cells and mesenchymal stromal cells sourced from umbilical cords. Researchers are also investigating non-invasive delivery techniques, such as intravenous injection, and are developing new treatment protocols that incorporate repeated dosing. Innovative cell products, including epicardial patches embedded with cardiomyocytes, are under evaluation, alongside cell-free therapeutic options like secretomes enriched with extracellular vesicles or exosomes.

The results of these trials will enhance our understanding of cell-based and cell product-based therapies as complementary additions to guideline-directed medical therapy for HF patients. Future research must address current barriers, refine treatment protocols, and ensure the scalability and reproducibility of these therapies. To improve perspectives on cardiology interventions, it is crucial to discuss the broader implications and future directions for cell therapy in HF.

Modern interventional cardiology is best understood as a continuum that begins with physiological lesion assessment and extends toward progressively advanced catheter-based therapies, rather than as isolated procedural domains (41, 48). Fractional flow reserve (FFR), instantaneous wave-free ratio (iFR), and complementary indices function as gatekeeping tools that quantify ischemic burden, residual risk, and the necessity for revascularization, thereby directly influencing downstream device selection and procedural intensity (41, 48). Landmark randomized trials such as FAME and FAME-2 demonstrated that physiology-guided PCI reduces unnecessary stent implantation while improving long-term clinical outcomes, firmly establishing coronary physiology as the foundation of modern PCI decision-making (41, 48). Within this framework, physiology-guided PCI informs not only *whether* to intervene, but also *how* aggressively to pursue lesion preparation, intravascular imaging, and stent optimization. The evolution of contemporary drug-eluting stent (DES) platforms characterized by thinner struts, biocompatible or bioresorbable polymers, and optimized drug kinetics has been closely aligned with insights gained from physiological assessment and imaging-guided deployment strategies. Previous reports emphasize that this integration of physiology and device technology has been central to reducing restenosis, stent thrombosis, and late adverse events in complex coronary anatomy (41, 48, 108, 109). The transition from coronary intervention to structural therapies such as transcatheter aortic valve implantation (TAVI) reflects a shared methodological and philosophical shift rather than a thematic discontinuity. Both PCI and TAVI rely on catheter-based delivery, high-resolution imaging, and avoidance of surgical morbidity in increasingly elderly and high-risk populations. Contemporary analyses highlight that coronary and structural interventions are converging disciplines unified by minimally invasive strategies, procedural planning with multimodality imaging, and multidisciplinary heart-team decision-making (104, 109). Framing TAVI as an extension of catheter-based cardiovascular therapy (113, 114) rather than a separate subspecialty creates a coherent narrative that mirrors real-world interventional practice. This convergence is increasingly reflected in training pathways, hybrid catheterization laboratories, and guideline documents that emphasize integrated coronary-structural assessment before valve intervention (41, 48, 108, 109, 114).

Critical comparison and Quantification of outcomes in complex PCI scenarios: While PCI in post–CABG patients is often described as technically complex, contemporary evidence demonstrates substantial heterogeneity in outcomes depending on target vessel selection. Large observational registries and meta-analyses consistently show worse long-term outcomes with saphenous vein graft (SVG) PCI compared with native-vessel PCI, including higher rates of distal embolization, periprocedural myocardial infarction, and target-vessel failure. Adjusted analyses from multicenter cohorts report approximately a 30–40% increase in major adverse cardiac events at 3–5 years following SVG PCI, despite modern DES use (115). These findings support a strategy favoring native-vessel PCI whenever anatomically feasible and underscore the importance of physiological and intravascular imaging guidance to identify ischemia-producing lesions and optimize outcomes in this high-risk population. IVUS- or OCT-guided PCI has been shown to significantly reduce target-vessel failure and stent thrombosis in complex anatomy, reinforcing its role as a standard of care rather than an adjunct (116). Randomized evidence comparing physiology-guided PCI with surgical revascularization has further refined expectations regarding FFR-based strategies. In the FAME-3 trial, FFR-guided PCI reduced the number of stents implanted but failed to achieve non-inferiority to CABG for the composite endpoint of death, myocardial infarction, stroke, or repeat revascularization at mid-term follow-up. This difference was primarily driven by higher rates of repeat revascularization and spontaneous myocardial infarction in the PCI group, despite similar mortality (117).

Although physiology-guided PCI offers clear advantages in lesion selection, its limitations are increasingly recognized in specific clinical contexts. FFR may underestimate ischemic risk in diffuse coronary disease, microvascular dysfunction, and post-CABG anatomy, where altered coronary hemodynamics and competitive flow reduce the accuracy of pressure-based indices. These limitations help explain the discordance between early FAME trials and later studies such as FAME-3, in which increasing anatomical complexity diminished the prognostic equivalence of PCI and CABG (118, 119). Future research should therefore move beyond binary comparisons of guidance strategies and toward integrated decision-making models that combine coronary physiology with plaque morphology and myocardial vulnerability (120, 121). Priority areas include randomized trials integrating FFR with IVUS or OCT in post-CABG patients, long-term comparative studies of drug-coated balloons versus DES in high-bleeding-risk populations, and standardized outcome definitions for emerging structural devices. Such targeted approaches address clearly defined evidence gaps rather than reiterating non-specific calls for further study (120, 121).

Transcatheter treatment of pure aortic regurgitation: Device-specific evidence: Transcatheter treatment of pure native aortic regurgitation (AR) presents unique challenges due to the absence of annular calcification required for anchoring conventional TAVI devices. Dedicated systems such as the JenaValve Trilogy and J-Valve have been engineered specifically for non-calcific AR using leaflet-engagement and locator-based fixation mechanisms. The pivotal ALIGN-AR study evaluating the JenaValve Trilogy demonstrated procedural success exceeding 95%, low early mortality, and sustained hemodynamic improvement, albeit with permanent pacemaker implantation rates approaching 20–25% at 1 year (122). Early multicenter experiences with the J-Valve system have reported similarly high technical success and favorable mid-term outcomes, reinforcing the importance of device-specific design in this setting. These results contrast sharply with earlier off-label TAVI experiences using standard devices, which were associated with valve embolization, residual regurgitation, and procedural instability. Collectively, these data support a paradigm shift toward dedicated transcatheter solutions for pure AR, while highlighting the need for long-term durability and comparative effectiveness studies (122, 123).

**FIGURE 5:**
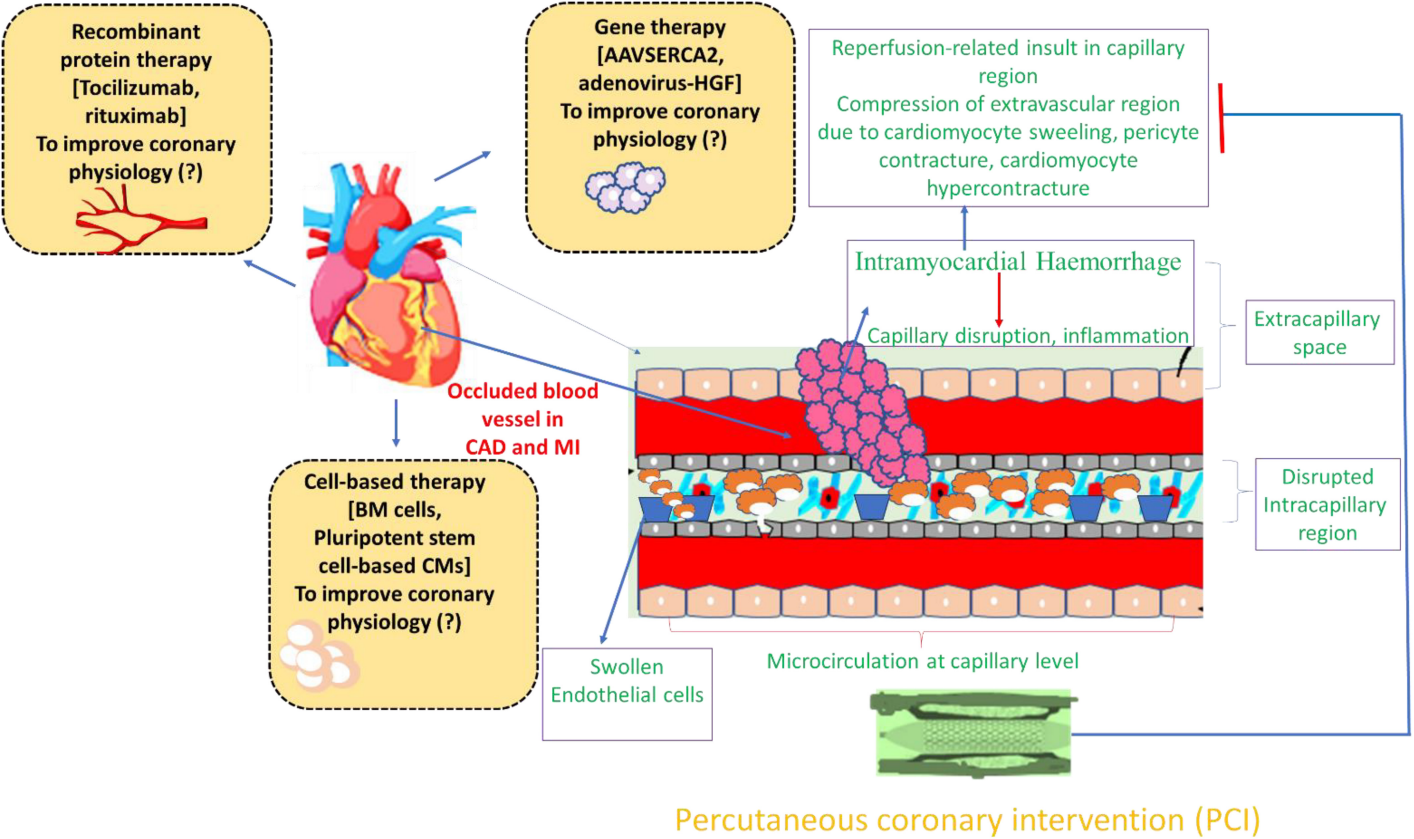
Pathophysiological mechanisms underlying microvascular obstruction and coronary no-reflow and the ameliorative strategies of cardiac ailments to improve coronary physiology. This figure depicts the complex pathophysiological mechanisms at play within the epicardial coronary arteries and the microcirculation (capillary) levels. The external forces that compress the microcirculation, subsequently cause a higher vascular permeability. Microvascular obstruction plays a pivotal role in the phenomenon of coronary no-reflow. Onset of this obstruction and no-reflow following reperfusion of an occluded coronary artery is driven by a combination of factors: myocardial ischemia, spontaneous or iatrogenic distal embolization, reperfusion injury, and individual susceptibility that heightens the likelihood of these events. These conditions frequently manifest after reperfusion therapy in patients experiencing ST-elevation myocardial infarction (STEMI). The prevalence of MVO and CNR after primary percutaneous coronary intervention (PCI) varies significantly based on the diagnostic tools used and the timing of the evaluation. Microvascular obstruction significantly diminishes the benefits of reperfusion therapy, leading to adverse clinical outcomes such as left ventricular remodeling, worsening or new onset heart failure, and increased mortality. Recognizing their critical impact on prognosis, patients with STEMI who develop microvascular obstruction require thorough risk assessment and customized therapeutic strategies. Furthermore, various elements contributing to microvascular obstruction including atherosclerotic debris, inflammasome. Additionally, the discussion addresses the barriers to approving biologics for cardiac diseases and anticipates future advancements. Monoclonal antibodies (mAbs), gene therapy, and cell therapy have shown significant promise in other medical fields, notably oncology, and hold potential for treating cardiac ailments. Research in CVDs has focused on inducing cardioprotection, promoting angiogenesis, inhibiting fibrosis, enhancing contractility, reducing major risk factors such as atherosclerosis, and stimulating cardiac regeneration, with promising results primarily in animal models.

Personalized approaches that leverage pharmacogenomics and patient-specific factors could optimize the outcomes of cell therapy. Identifying biomarkers for patient selection will be essential in maximizing therapeutic benefits. For instance, integrating cell therapy with existing treatments, such as drug regimens and mechanical support devices, could offer synergistic effects, potentially improving patient outcomes more than any single therapy alone. Long-term follow-up and monitoring of patients undergoing cell therapy are necessary to understand the durability of these treatments and their impact on overall cardiac function and quality of life. In addition, addressing regulatory and ethical issues surrounding cell therapy, including the use of stem cells and genetically modified cells, will be crucial for the broader acceptance and implementation of these therapies. Advances in bioengineering and regenerative medicine, such as the development of bioengineered heart tissues and organoids, could pave the way for more effective and targeted cell therapies. Yet, the journey of cell therapy for HF is marked by significant trials and challenges but continues to evolve. Ongoing research and clinical trials hold the promise of making cell-based therapies (Figure 5) a viable and effective treatment option for HF, complementing existing medical practices. By integrating personalized medicine, combining therapies, and addressing long-term efficacy and ethical considerations, the future of HF treatment looks promising, with great potential for innovation and improved patient outcomes (112).

## 12 Conclusion and future acknowledgments

Advancements in coronary interventions, particularly the integration of cell-based therapies, have shown promising potential in treating heart failure and CAD. The exploration of new cell types, delivery methods, and treatment protocols has paved the way for significant improvements in patient outcomes. Clinical trials over the past two decades have described safety and efficacy of various cell-based treatments, reinforcing their role as a complementary approach to existing medical therapies. Despite initial skepticism and numerous challenges, the field has made substantial progress, offering new hope for patients with limited treatment options. The translation of preclinical findings into clinical practice has underscored the importance of personalized medicine and the need for a multidisciplinary approach in managing HF and CAD. The inclusion of pharmacogenomics in patient selection and the combination of cell therapies with traditional treatments could enhance therapeutic efficacy and patient quality of life. Moreover, the integration of bioengineering advancements, such as bioengineered heart tissues and organoids, holds potential for even more targeted and effective interventions. Ongoing and future clinical trials should continue to investigate novel cell types, such as pluripotent stem cell-derived cardiomyocytes and umbilical cord-derived mesenchymal stromal cells. Additionally, exploring innovative delivery methods, such as intravenous injections and epicardial patches, will help optimize the administration of cell-based therapies.

Addressing disparities in healthcare delivery and making cell-based therapies affordable and accessible to diverse populations will be critical for the widespread adoption of these treatments. The evolving landscape of coronary interventions, driven by advancements in cell therapy and regenerative medicine, holds immense promise for the future of cardiovascular care. By embracing personalized medicine, exploring combination therapies, and leveraging technological innovations, we can significantly improve patient outcomes and quality of life. Continued research, collaboration, and ethical considerations will be essential in realizing the full potential of these groundbreaking therapies, paving the way for a new era in the treatment of coronary artery disease and heart failure.

## Novelty statement

This review provides an integrated, critical synthesis of contemporary advances in PCI, emphasizing how coronary physiology, imaging, and device innovation collectively inform modern revascularization strategies. By addressing conflicting evidence, clinical limitations, and emerging technologies, the review offers actionable insights for optimizing PCI in complex coronary artery disease.
